# Feasibility of genetic testing for cancer risk assessment programme in Nigeria

**DOI:** 10.3332/ecancer.2021.1283

**Published:** 2021-09-07

**Authors:** Prisca O Adejumo, Toyin I G Aniagwu, Olutosin A Awolude, Abiodun O Oni, Olubunmi O Ajayi, Omolara Fagbenle, Dasola Ogungbade, Makayla Kochheiser, Temidayo Ogundiran, Olufunmilayo I Olopade

**Affiliations:** 1 Department of Nursing, College of Medicine, University of Ibadan, Ibadan, 200284, Nigeria; 2 School of Occupational Health Nursing, University College Hospital, Ibadan, 200212, Nigeria; 3 Department of Obstetrics and Gynaecology, College of Medicine, University of Ibadan/University College Hospital, Ibadan, 200284, Nigeria; 4 Department of Surgery, University College Hospital, Ibadan, 200212, Nigeria; 5 Department of Radiation Oncology, University College Hospital, Ibadan, 200212, Nigeria; 6 Department of Radiology, University College Hospital, Ibadan, 200212, Nigeria; 7 Center for Clinical Cancer Genetics, University of Chicago, Chicago, IL, 60637, USA; 8 Center for Global Health, University of Chicago, Chicago IL, 60637, USA

**Keywords:** breast cancer, ovarian cancer, genetic counselling, genetic testing, genetic risk assessment

## Abstract

**Background:**

A high frequency of BRCA mutations has been established in Nigerian breast cancer (BC) patients. Recently, patients’ and first-degree relatives’ interest have been raised on cancer genetic risk assessment through our awareness activities in Nigeria. This led to the emergence of nurse-led cancer genetic counselling (CGC) and testing aimed at providing standard-of-care for individuals at increased risk of hereditary breast and ovarian cancers.

**Methods:**

In June 2018, CGC and testing of patients with BC and ovarian cancer (OC) commenced in collaboration with Color Genomics Inc. for a 30-panel gene testing. Previously trained nurses in CGC at the University College Hospital, Ibadan offered genetic counselling (GC) to willing patients with BC and gynaecological cancer in four out-patient oncology clinics and departments for the pilot study. Consultation consisted of CGC, patient’s history, pedigree and sample collection for genetic testing (GT).

**Results:**

Forty-seven patients – 40 with BC, five with OC and two with endometrial cancer received GC, and all chose to undergo GT. The average age at testing was 48.2 ± 12.1 years. Eight women reported a known family cancer history and there were more perceived beneﬁts than barriers to GT with the patients experiencing the desire for none of their relative to have cancer. Results revealed no mutations in 27 (57.4%), 16 (4.0%) variants of unknown signiﬁcance and 4 (8.5%) pathogenic mutations.

**Conclusion:**

Personalised cancer care utilises GC and testing for cancer risk assessment towards prevention and early detection in high risk women. The study indicates the necessity of expanded cancer genetic services for integration into patient care and cancer prevention.

## Introduction

Hereditary cancer syndromes account for approximately 5%–10% of all cancers [1, 2] with breast cancer (BC) and ovarian cancer (OC) association with germline mutations established since the early 1990s [3]. Hereditary breast and ovarian cancer (HBOC) is a syndrome particularly affecting certain populations [4] like the African-American women [5] and African women [6, 7]. Studies in Nigeria, the most populous African country, have reported a high frequency of germline mutations, particularly of BRCA1/2 variant associated with HBOC risk, in the Nigerian women of up to 16% [8, 9]. The implication is that many Nigerian women have up to an 85% and 46% lifetime risk of BC and OCs, respectively. While BC is the leading cause of cancer death in women in Nigeria, OC incidence is increasing and remains the most fatal of all gynaecological cancers [10, 11] particularly due to late presentation of these cancer cases [12, 13] associated with absent effective methods for screening and early diagnosis.

Evaluation of the likelihood of a patient having one of these cancer predisposition syndromes enables physicians to provide individualised assessments of cancer risk, as well as the opportunity to provide tailored screening and prevention strategies such as surveillance, chemoprevention and prophylactic surgery that may reduce the morbidity and mortality associated with these syndromes [14]. This is especially so now that genetic risk assessment is rapidly becoming an expectation in oncology care [15, 16]. A review of service models for provision of genetic healthcare [17] highlighted the effective use of multidisciplinary clinics and services to ensure that patients and families have access to this coordinated care. However as highly proposed and used, BC and OC prevention have not explored this novel opportunity in low- and middle-income countries like Nigeria [18]. There is low awareness of cancer genetics in Nigeria [18] and resources for risk assessment and communication have been lacking. This has led to a growing demand in knowledge for genetic testing (GT) services [19]. The establishment of a cancer risk assessment programme therefore will bridge the knowledge gap about cancer genetics between health professionals and patients [20]. Genetic counseling (GC) and subsequent testing for deleterious gene mutations impacts psychosocial assessment and support, derivation of personalised risks and the likelihood of identifying a mutation with genetic susceptibility testing [14]. This will increase family understanding of testing options and ensure that the most appropriate test is ordered, allowing for informed decision making, and ensuring that families are prepared for the outcomes of testing [21].

Enquiries are made by patients and their relatives on cancer genetic risk assessment through our recent awareness activities on cancer genetics in Nigeria. This led to the emergence of a nurse-led cancer genetic counselling (CGC) and testing. This study aimed to explore the feasibility of GT for risk assessment programme in Nigeria in making CGC and testing standard-of-care for individuals at increased risk of HBOCs by introducing the services to cancer care at the University College Hospital, Ibadan.

## Methods

We conducted a pilot study testing the feasibility of integrating systematic cancer risk assessment and GC as a standard and routine component of oncology management at the University College Hospital (UCH) , Ibadan, Nigeria between July and August 2018. Nurses who received a 1 week abridged intensive training in CGC at the UCH, Ibadan in 2014–2015 offered GC to willing patients with BC, OC and endometrial cancer, but without prior genetic counselling and testing (GCT) in four out-patient oncology clinics and departments of the hospital. The nurses’ training was based on the Cancer Genetics and Risk Management training which the lead nurse and the coordinating nurse had received from the University of Iowa, The University of Chicago and the City of Hope, United States of America from 2013 to 2016. Counselling and interview session discussions included educating about genetics of hereditary breast and gynaecological cancers, beneﬁts and risks of GC; documentation of personal and family history with pedigree drawing; determination of cancer mutation risk, GT methods and meaning of results. Consenting patients were, subsequently, tested with 30 cancer susceptibility gene panel in the Color® Genomics kit which include **BAP1, MITF, CDK4, CDKN2A, ATM, CDH1, NBN, CHEK2, PTEN, BRCA1, BRCA2, PALB2, BARD1, BRIP1, TP53, STK11, MLH1, MSH2, EPCAM, MSH6, PMS2, RAD51C, RAD51D, APC**, BMPR1A, SMAD4, GREM1, MUTYH, POLD1, POLE using targeted sequencing panel. Sequencing was done on an Illumina NextSeq 500/550 instrument for 150 bp paired-end sequencing. The genetic variants were reviewed, discussed and classified as likely pathogenic, or pathogenic and variants of uncertain significance, according to the American College of Medical Genetics and Genomics 2015 guidelines.

After testing, patients completed a semi-standardised questionnaire assessing their socio-demographic information, family cancer history and perceived beneﬁts and barriers to GT. Ethical approval for the study was obtained from the University of Ibadan (UI)/UCH Ethics Committee with the number UI/EC/18/0251. Institutional support was obtained, based on the awareness previously raised on CGC.

## Results

Forty-seven women with cancers (BC = 40 (85.1%), OC = 5 (10.0%) and endometrial = 2 (4.3%)) who consented to participate were recruited from the oncology clinics and departments of the selected hospital. The women received GC and subsequently, had GT. The mean age at the time of testing was 48.2 (± 12.1 years; Range: 28–70) years. Family history of cancer was reported by eight (17.0%) of the women (Table 1).

Perceived personal risk of cancer recurrence and the lifetime risk of their relatives showed that 42.8% believed that they cannot have cancer again while 17.2% agreed to a risk of 50% and above. A considerable proportion of participants had a view that their children (24.0%), siblings (25.5%) and parents (13.5%) have above 50% risk of developing cancer (Table 2).

More participants (75.6%) had concerns about their families developing cancer and they identified such as not wanting their relatives to develop cancer like them (67.6%) and desiring that the relatives will know how cancer can be prevented (Table 3). To this effect, most of the participants (93.5%, 91.3% and 93.3%) would like their relatives to have GC, discuss their risks with a specialist and undergo GT, respectively (Table 3).

GT for risk assessment was perceived by the participants to be beneficial Top three of their perceived benefits of cancer GCT were, cancer prevention (89.4%), early detection of cancer (70.2%), motivation for self-examination (61.7%). The most mentioned barriers to GC and testing services were cost (80.9%), accessing testing centres (55.3%) and availability of test (38.9%) (Table 4).

**Figure 1.**
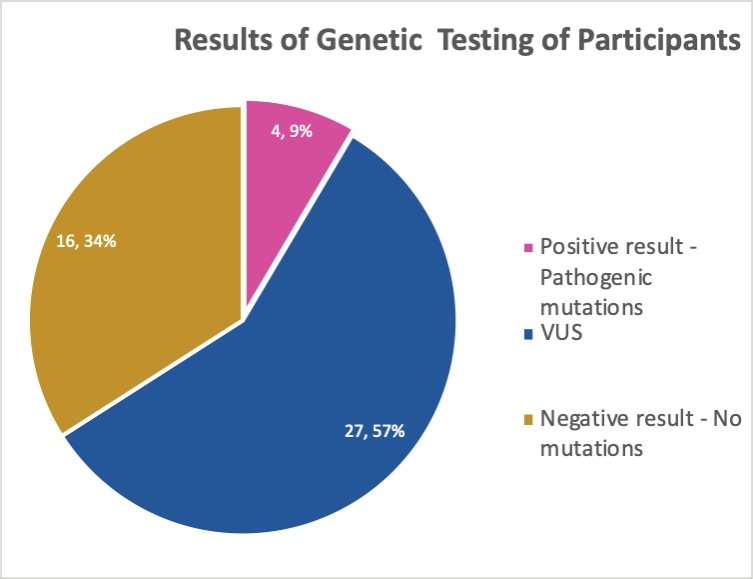
Results of GT undergone by the participants.

All the participants planned to disclose and discuss their test results with their relatives who were mostly children (Daughters – 72.3%, sons – 68.1%) and siblings (sisters – 74.5%, brothers 55.3%) as shown in Table 5. As all the participants tested for genetic mutations, the result showed about 9.0% pathologic gene mutation which were in BRCA 1, BRCA 2 and ATM, negative result showing no mutations were 34.0% while more than half, 57.0% turned out to be variance of uncertain significance (VUS) (Figure 1). Details of the mutations are as follows: BRCA 1 variant c.5095C>T (p.

Arg1699Trp) alternate names, g.41215948G>A, BIC: R1699W; BRCA 2 c.7900delA (p.Met2634Trpfs*14) alternate names, g.32936754delA, BIC: 8128delA; ATM variant c.1066-2A>T, alternate name, g.108119658A>T, ATM variant c.72+1G>A alternate name, g.108098424G>A. All mutations are of heterozygous.

## Discussion

This study set out to explore the feasibility of an integrated CGC and testing in the care of individuals with cancers and their hereditary cancer at-risk relatives in UCH, Nigeria. With the advent of more targeted and personalised approaches to cancer prevention and treatment, it has become imperative to understand the genetic basis of BC and gynaecological cancer such as OC and endometrial cancer. This is vital in providing patients with the needed effective preventive and/or management strategies towards improvement of outcomes. The causes of hereditary susceptibility to some women cancers have been documented to include hereditary cancer susceptibility genes, BRCA1 or BRCA2, associated with HBOC syndrome [22, 23], DNA mismatch repair genes, MLH1, MSH2, MSH6, or PMS2, in Lynch/Hereditary Non-Polyposis Colorectal Cancer in endometrial, colorectal and OC [24, 25], Cowden syndrome in endometrial and BC [26, 27] and Li-Fraumeni syndrome in BC [28, 29]. In this study, the patients’ personal and relatives’ perceived lifetime cancer risk was explored. The perceived personal risk of developing cancer of 17.2% is a pointer to the fact that the belief system still plays a role in the aetiology of the disease as cancer is generally perceived as a taboo among the people of African descent [30, 31]. This further shows a gap that needs to be filled by providing appropriate cancer genetic risk assessment education and cancer care practice in this era of personalised care. However, 63% concerns the women communicated in the study about their relatives developing cancer is an indication for their ardent need for interventions in the area of CGC and service provision. This has also been shown in studies that positive perceptions of the public towards GT and its beneficial function in the healthcare as a factor in its uptake [32, 33]. This understanding of patients’ perceived risk is salient to the establishment of cancer genetic risk assessment and management services as the risk of a second primary BC in *BRCA* 1 or 2 mutation carriers, particularly those diagnosed with BC at a younger age, is much higher (upwards of 50%) than in non-carriers [34]. Also, with this knowledge, it is now paramount to identify women who carry mutations which can lead to the utilisation of the targeted medical advances in prevention, early detection and treatment [35, 36].

With the evidence that 5%–10% of BCs and 10%–15% of OCs are hereditary [37], the result of gene testing from this pilot study is not implausible. Moreover, up to 16% germline mutations have been reported in the Nigerian women with BC [9, 38]. The authors believe that with the concerns of the patients about their relatives; possibility of getting cancer, the pooling of multiple generations with BC, OC and other related cancers is possible. Efforts at cancer prevention and early detection have of recent been expanded towards pre-cancer/pre-symptomatic interventions [36]. These can be tailored to individual care given to women at increased risk for hereditary breast and gynaecological cancers.

A positive inclination in the patients’ perceptions on the benefits of cancer genetic assessment services especially to their relatives was also noted. Studies found that members of families with identified *BRCA* 1 and 2 mutations were more likely to have GT when the genetic test results are shared [39, 40]. In these families, cancer-specific distress and worry play a significant role in the choice to test for *BRCA1* mutations as does a greater perceived risk of being a mutation carrier and of developing BC or OC, and the perception that the advantages of *BRCA* testing outweigh the disadvantages [41]. The family serves as a vital communication nexus for information exchange [42] and may be an avenue for sharing information on cancer risk and prevention strategies. The intention to disclose and discuss the genetic test result to close relatives such as siblings and children is an indication of the assertion in other studies [40, 42].

Cancer risk assessment and associated GT are essential services in cancer risk prevention and are therefore important to be integrated to cancer care. This is, therefore, a right step in the right direction as the findings indicate its feasibility. This is crucial for a greater degree of personalised and yet comprehensive cancer care including GT for cancer risk assessment programme in Nigeria.

### Limitation of the study

There is need for more data on a larger population for increased level of generalisation. Color Genomics® is the only laboratory engaged in this study which although have variants tested for African American population, is being engaged for the first time in the Nigerian population.

However, multi gene panels including Color Genomics® 30 gene panel have been used extensively among African Americans. This advancement in sequencing panels for hereditary BC, OC and prostate cancer has shown that people of African descent tend to have more rare multiple variants and VUS than Caucasians [43, 44]. Also, Color Genomics® 30 gene panel has been used among Africans in Uganda and Cameroon [7]; therefore, this study relies on the result to provide population relevant data.

## Conclusion

Cancer GC and testing are perceived by patients in this study as beneficial for providing risk assessment for personalised patient care, early detection and prevention in women with high BC’s and gynaecological cancers’ risk. These results are important as GC and testing are expected to be offered to newly diagnosed BC, OC and endometrial cancer patients with increasing frequency in order to inform these women and their relatives about the possibility of a familial/hereditary nature of their disease to influence both their treatment and prevention for their family members. Proper education of the patients and their relatives on cancer genetic risk management will facilitate the required attention for maximal utilisation of cancer genetic services.

## Authors’ disclosures of potential conflicts of interest

Olufunmilayo I Olopade is an equity stock holder of CancerIQ. The other authors declare no conflicts of interest.

## Funding declaration

This project was supported by National Institutes of Health grants from Susan G. Komen for the Cure (OIO) and Breast Cancer Research Foundation (OIO) the Color Genomics® Foundation.

## Figures and Tables

**Table 1.** Participants’ sociodemographic characteristics *N* = 47.

Characteristics	Frequency	%
**Type of cancer** Breast Ovarian Endometrial	40 5 2	85.1 10.6 4.3
**Gender** Female	47	100.0
**Marital status** Single Married Separated/divorced Widowed	1 36 3 7	2.1 76.5 6.4 14.9
**Religion** Christianity Islamic	35 12	74.5 25.5
**Ethnicity** Yoruba Ibo Others	33 9 5	70.2 19.1 10.6
**Highest educational qualification** Elementary Secondary Diploma /National Certificate of Education B.Sc. M.Sc. Ph.D.	7 7 13 (27.7) 17 (36.2) 2 (4.3) 1 (2.1)	14.9 14.9 27.7 36.2 4.3 2.1
**Any family had cancer** Yes No	8 39	17.0 83.0
Mean age: 48.2 ± 12.1 years; Range: 28–70 years		
Mean income: N51,542 ± N46,007; ($136 ± 121.07) Range: N2,000–N180,000 (Equivalent to – $474)		

**Table 2.** Participants’ perceived risk of personal and relative cancer risk.

Perceived lifetime risk of relatives developing cancer (0 is the lowest perceived risk, 10 highest perceived risk)	Frequency	%
**Risk of developing cancer again *n* = 35** 0 1 2 3 5 7 8 10	15 1 8 5 2 1 2 1	42.8 2.9 22.8 14.3 5.7 2.9 5.7 2.9
**Lifetime risk of children *n* = 25** 0 1 2 3 4 5 6 8	16 5 5 2 3 2 1	4.0 24.0 20.0 20.0 8.0 12.0 8.0 4.0
**Lifetime risk of any siblings *n* = 43** 0 1 2 3 4 5 6 8 9	17 5 5 4 1 8 1 1 1	39.5 11.6 11.6 9.3 2.3 18.6 2.3 2.3 2.3
**Lifetime risk of any of parents *n* = 37** 0 1 2 3 4 5 6 9	18 5 5 2 2 2 1 2	48.6 13.5 13.5 5.4 5.4 5.4 2.7 5.4

**Table 3.** Participants’ concerns about their relatives’ risks of cancer.

Concerns about relatives’ cancer risk	Frequency	%
**Do you have concern about other relatives getting cancer *n* = 45** Yes No	34 11	75.6 24.4
**Concerns *n* = 34** I don’t want them to develop cancer I will like them to know that cancer can be prevented	23 11	67.6 32.4
**Will you like for your relative to have GC? *n* = 46** Yes No	43 3	93.5 6.5
**Will you like your relative to discuss their risks with a specialist *n* = 46** Yes No	42 4	91.3 68.7
**Will you like your relatives to have GT *n* = 45** Yes No	42 3	93.3 6.7

**Table 4.** Participants’ perceived benefits and barriers of GT *N* = 47.

Perceived benefits	Frequency	%
Motivate self-exam	29	61.7
Helps family and children	25	53.2
Reduces concern about cancer	24	51.1
Reduces uncertainty	20	42.5
Provides sense of personal control	22	46.8
Helps plan the future	27	57.4
Helps make important life decisions	24	51.1
Helps with cancer prevention	42	89.4
Early detection of BC	33	70.2
**Perceived barriers**		
Cultural perception	11	23.4
Cost	38	80.9
Access to the testing centre	26	55.3
Availability of test	18	38.3
Anticipated worry about offspring/relative if result is positive	13	27.7
Anticipated personal emotion if result is positive	18	38.3
Worry that other would find out	12	25.5
Time	7	14.9
Not wanting blood taken	9	19.1
Lack of interest	9	19.1
Worry about increased risk	10	21.3
Worry about discomfort	7	14.9

**Table 5.** Relatives that participants would discuss result of GT with.

Relatives to discuss result with		Frequency	%
Father		7	14.9
Mother		17	36.2
Brother(s)		26	55.3
Sister(s)		35	74.5
Daughter(s)		34	72.3
Son(s)		32	68.1
Spouse		18	38.3
Others	Step parents	1	2.1
	Aunts	8	17.0
	Uncles	2	4.3
	Cousin(s)	3	6.4
	Daughters-in-law	1	2.1
